# Emodin Protects Sepsis Associated Damage to the Intestinal Mucosal Barrier Through the VDR/ Nrf2 /HO-1 Pathway

**DOI:** 10.3389/fphar.2021.724511

**Published:** 2021-12-20

**Authors:** Luorui Shang, Yuhan Liu, Jinxiao Li, Guangtao Pan, Fangyuan Zhou, Shenglan Yang

**Affiliations:** Department of Integrated Traditional Chinese and Western Medicine, Union Hospital, Tongji Medical College, Huazhong University of Science and Technology, Wuhan, China

**Keywords:** emodin, cecal ligation and puncture, lipopolysaccharide, intestinal barrier dysfunction, VDR/ Nrf2 /HO-1 signaling pathway

## Abstract

**Aims:** Emodin is an anthraquinone extracted from Polygonum multiflorum, which has potential anti-inflammatory and anti-oxidative stress effects. However, the possible protective mechanism of emodin is unclear. The purpose of this study was to investigate the protective mechanism of emodin against cecal ligation and puncture and LPS-induced intestinal mucosal barrier injury through the VDR/ Nrf2 /HO-1 signaling pathway.

**Methods:** We established a mouse model of sepsis by cecal ligation and puncture (CLP), and stimulated normal intestinal epithelial cells with lipopolysaccharide (LPS). VDR in cellswas down-regulated by small interfering ribonucleic acid (siRNA) technology.Mice were perfused with VDR antagonists ZK168281 to reduce VDR expression and mRNA and protein levels of VDR and downstream molecules were detected in cells and tissue. Inflammation markers (tumor necrosis factor-α (TNF-α), interleukin-6 (IL-6)) and oxidative stress markers (superoxide dismutase (SOD), malondialdehyde (MDA) and glutathione (GSH)) were measured in serum and intestinal tissueby enzym-linked immunosorbent assay. The expression of VDR in intestinal tissue was detected by immunofluorescence. Histopathological changes were assessed by hematoxylin and eosin staining.

**Results:** In NCM460 cells and animal models, emodin increased mRNA and protein expression of VDR and its downstream molecules. In addition, emodin could inhibit the expressions of TNF-α, IL-6 and MDA in serum and tissue, and increase the levels of SOD and GSH. The protective effect of emodin was confirmed in NCM460 cells and mice, where VDR was suppressed. In addition, emodin could alleviate the histopathological damage of intestinal mucosal barrier caused by cecal ligation and puncture.

**Conclusion:** Emodin has a good protective effect against sepsis related intestinal mucosal barrier injury, possibly through the VDR/ Nrf2 /HO-1 pathway.

## Introduction

Sepsis is a complex condition of organ dysfunction caused by a host’s dysfunctional response to infection. (Shankar-Hari et al., 2016) Although studies have been conducted on drugs to treat sepsis, the disease still has a high mortality rate. (Condotta et al., 2013) This is a challenge that needs to be faced and overcome clinically. (Cao et al., 2018) Previous studies have indicated that the intestinal mucosal barrier dysfunction in sepsis with multiple organ dysfunction syndrome in plays an important role. (Yoseph et al., 2016; Assimakopoulos et al., 2018; Xu et al., 2018) Biologically, the intestinal mucosal barrier prevents intestinal microbes and their products from entering the bloodstream from the gut, When sepsis occurs, excessive inflammation damages the intestinal epithelial cells, leading to damage to the intestinal mucosal barrier, which promotes bacterial migration and toxin transmission. (Guthrie et al., 2015; Hoeger et al., 2017) Harmful substances further activate host immune defense mechanisms, leading to multiple organ failure and life-threatening clinical symptoms. The gut is considered the ‘motor’ of sepsis and multiple organ dysfunction syndrome (MODS). (Ramírez, 2013; Wang et al., 2017) Therefore, there is an urgent need for safe and effective treatment strategies for sepsis associated models of intestinal mucosal barrier injury. It is essential to improve the prognosis and survival of patients with sepsis. (Chen et al., 2018)

**GRAPHICAL ABSTRACT:**
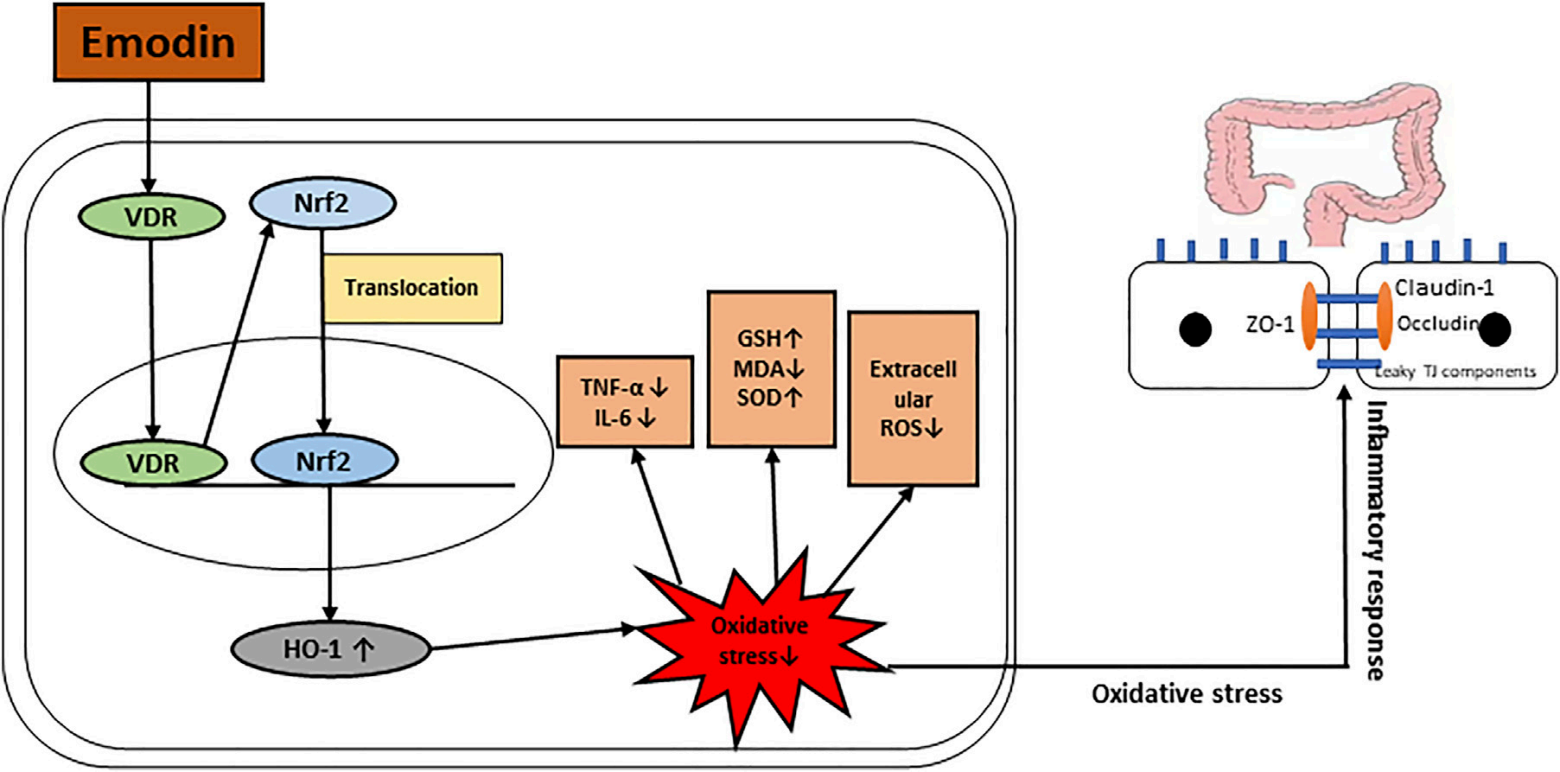
Possible mechanism of how emodin improves intestinal mucosal barrier.

Vitamin D receptor is a nuclear hormone receptor that is highly expressed in the intestine. Studies have shown that the VDR pathway protects the intestinal barrier mainly through up-regulation of tight junction proteins and regulation of innate immune response. (Kong et al., 2008; Liu et al., 2013; Chen et al., 2015; Meckel et al., 2016) In addition, the tight junction protein Claudin-2 is a direct target of the transcription factor VDR. (Zhang et al., 2015) VDR signaling pathway also has anti-inflammatory, anti-oxidative stress, anti-apoptosis and other functions. (Xu et al., 2015; Du et al., 2019; Kellermann et al., 2020) Therefore, VDR plays an important role in maintaining the integrity of the intestinal mucosal barrier.

Nuclear factor erythroid 2-related factor (Nrf2) is a key regulator of oxidative stress and inflammation. Under oxidative stress, activated Nrf2 is transferred from the cytoplasm to the nucleus, then, it can mediate the transcription of target genes and regulate downstream antioxidant enzymes, such as heme oxygenase-1 (HO-1), SOD, MDA, GSH, to reduce oxidative stress damage. (Shah et al., 2017) However, there are few studies on the relationship between VDR and Nrf2.Emodin has a wide range of pharmacological functions, including anti-inflammatory, anti-oxidation, anti-tumor and so on. (Wang et al., 2015; Tian et al., 2018)Of course, previous studies have shown that emodin ameliorates LPS-induced damage to intestinal epithelial barrier function. (Lei et al., 2014) However, the potential mechanism of emodin on intestinal barrier function is not fully understood. In this study, we hypothesized that emodin could reduce the production of pro-inflammatory cytokines, increase the levels of antioxidant factors, and ameliorate sepsis related intestinal mucosal barrier damage through the VDR/ Nrf2 /HO-1 signaling pathway. Based on this assumption, we conducted the following studies.

## Materials and Methods

### Reagents

#### Cell Culture and Reagents

NCM460 Cell lines were purchased from Shanghai Cell Collection (Catalog No:AC339288) and cultured in Dulbecco’s Modified Eagle’s Medium (DMEM; Gibco, Catalog No:11965092) supplemented with 10% fetal bovine serum(FBS; Gibco, Catalog No:10100147), 100 units/mL of penicillin and 100 units/mL of streptomycin(Solarbio, Catalog No: P1400). The medium should be changed every 2 days. The cells were placed in a humidification chamber at 37°C and 5% CO2. Emodin (Catalog No:B7881), LPS (Catalog No:L2880) and DEM(Catalog No:D4902) were purchased from Sigma-Aldrich. A Cell Counting Kit-8 reagent (CCK-8)( Catalog No:C0037) was obtained from Beyotime. The TNF-α (Catalog No:FEK0527) and IL-6 (Catalog No:EK0411) ELISA kits were obtained from BDR Biology (Wuhan, China). The antibodies used were as follows: Anti-mouse VDR (Catalog No:sc-13133) was purchased from Santa Cruz Biotechnology. Anti-ZO-1 (Catalog No:A0659), anti-Occludin (Catalog No:A2601), anti-Claudin-1 (Catalog No:A2196), anti-GAPDH (Catalog No:AC002) were obtained from ABclonal Technology (Wuhan, China). Anti-Nrf2 (Catalog No:ab62352), anti-phospho-Nrf2 (Catalog No:ab76026), anti-HO-1 (Catalog No:ab52947), anti-GAPDH (Catalog No:ab8245) were provided from Abcam (Abcam, Cambridge, MA, United States). Horseradish peroxidase-conjugated goat anti-mouse(Catalog No:4741506) and anti-rabbit(Catalog No:4741806) species-specific secondary antibodies were provided from CW Bio, Co., Ltd.

### Cytotoxicity of Emodin

The cytotoxicity of emodin was assessed by CCK8 assay. NCM460 cells were inoculated with 105 cells per well and cultured overnight in 96-well plates. Then, emodin with different concentration gradients (3.75, 7.5, 15, 30, 60, 120, 240 μg/ml) was added. (Yang et al., 2017) After 24 h, 10 ml CCK8 solution was added to each well and kept away from light for 2 h. Finally, the absorbance values of all the holes were read at 450 nm with a microplate reader. In addition, the experiment was carried out three times, and the results were expressed by means ± SEM. The cell survival rate was calculated according to the following formula: cell viability (%) = (OD_blank_- OD_experiment_)/OD_blank_×100%. The procedure followed the previously published steps. (Xiong et al., 2019)

### Cellular Model Establishment and Intervention

NCM460 cells were divided into six groups, which were normal group, model group, emodin groups of different concentrations (15, 30, 60 μg/ml) and dexamethasone group(DEX) (0.5 g/ml, diluted in PBS medium). Cells were subcultured in 6, 12 or 96-well plates and cultured to 70% density. The blank group was cultured in the medium without any treatment and served as a negative control. Only LPS (1 g/ml) was added to the model group, and corresponding concentrations of solution were added to the emodin group and DEX as mentioned above. (Ding et al., 2018) After 24 h, the cells were collected for quantitative real-time polymerase chain reaction and Western blot analysis.

### Small Interfering RNA Transfection in NCM460 Cells

Small interfering RNA (si-RNA) (sense5′-CCCACCUGGCUGAUCUUGUCAGUUA-3′, antisense 5′-AAU​GGC​UUC​AAC​CAG​CUU​AGC​AUC​C-3′) was aynthesized by Oligobio (Beijing, China). According to the manufacturer’s instructions, NCM460 cells were cultured into a 6-well plate and transfected with 20 pmol/ ml Lipofectamine 2000(Invivogen,#11668030) and 2.5 pmol/ ml siRNA double stranded body. The medium was replaced 6 h after siRNA transfection. The cells were then cultured for another 48 h. The experimental group was treated with emodin or DXM 24 h before harvest.

### Animals

Forty-eight BALB /c SPF 8 week-old male mice, with a mass of 18–22 g, were purchased from Sibeifu (Beijing) Biotechnology Co., Ltd. (Animal License No. : SYXK (Hubei) 2019-0010). The experimental process was strictly carried out in accordance with the Guidelines for Ethical Review. Mice were routinely raised in SPF environment at 18–23°C, light with natural circadian rhythm, humidity of 55%–65%, and free intake of food and water. (Tsao et al., 2010)

### Establishment of the Cecal Ligation and Puncture Model

The model of polymicrobial sepsis was induced by CLP. (Rittirsch et al., 2009) In short, anesthesia was induced by intravenous administration of ketamine (80 mg/kg) and serazine (10 mg/kg). A 3 cm incision was made in the middle of the abdomen of mice, and the cecum was separated. The cecum was ligated at one-third of the distal cecum with a 3-0 suture. Two holes were pricked with a No. 21 needle, and a small amount of feces was excreted by extruding the punctured cecum. Sham surgery was also performed, but the cecum did not require ligation and perforation. The animals were resuscitated immediately after the operation by a subcutaneous injection of 1 ml of Ringer’s fluid. All experimental operations have been approved by the Laboratory Animal Ethics Committee of Tongji Medical College of Huazhong University of Science and Technology.

### Animal Grouping and Treatment

Forty-eight mice were randomly divided into six groups: sham operation group, model group, emodin low, medium and high concentration groups (20, 40, 80 mg/kg/day) and DEX group (1.8 mg/kg/day). In addition, the other 64 mice were divided into sham operation group and negative control antagonist group (NC antagonist group), NC ZK group, ZK + Model, ZK + Model supplemented with low, medium and high flavin groups (20, 40, 80 mg/kg) and DEX group. Mice were fed adaptively for 1 week, followed by 5 days, emodin group and DEX group were given the corresponding concentration of drug gavage every day. Mice were administered with 100 μl of vehicle, ZK(MedChemExpress, HY-12407) dissolved in oil (sunflower oil, Auchan). Each mouse was injected 1ug/kgZK for five consecutive days. Overnight fasting was performed before surgery and the model was established using the CLP method described above. The mice were sacrificed 48 h after operation. Samples and intestinal tissue were taken from all groups of mice and serum was separated by centrifugation.

### Reverse Transcription Quantitative Polymerase Chain Reaction

Total RNA was extracted from intestinal tissue using chloroform and Trizol (Invitrogen). The first cDNA template was synthesized using the primer reverse transcription kit according to the manufacturer’s instructions. Then, quantitative polymerase chain reaction was performed using SYBR premix kit (Vazyme, Q221-01). The relative number of transcripts was calculated according to the 2^−△△Ct^ formula. All RT-PCR was performed at least three times. All primers were designed by Primerbank, verified by NCBI/BLAST, and synthesized by GenScript(Nanjing, China). Table 1 provides the sequence of polymerase chain reaction primers.

**TABLE 1:** Primer sequence.

Gene name		Primer sequence(5’ to 3’)
VDR	Forward	GCT​CAA​ACG​CTG​CGT​GGA​CAT​T
	Reverse	GGA​TGG​CGA​TAA​TGT​GCT​GTT​GC
Nrf2	Forward	CAG​CAT​AGA​GCA​GGA​CAT​GGA​G
	Reverse	GAA​CAG​CGG​TAG​TAT​CAG​CCA​G
HO-1	Forward	CAC​TCT​GGA​GAT​GAC​ACC​TGA​G
	Reverse	GTG​TTC​CTC​TGT​CAG​CAT​CAC​C
ZO-1	Forward	GTT​GGT​ACG​GTG​CCC​TGA​AAG​A
	Reverse	GCT​GAC​AGG​TAG​GAC​AGA​CCA​T
Occludin	Forward	TGG​CAA​GCG​ATC​ATA​CCC​AGA​G
	Reverse	CTG​CCT​GAA​GTC​ATC​CAC​ACT​C
Claudin-1	Forward	GGA​CTG​TGG​ATG​TCC​TGC​GTT​T
	Reverse	GCC​AAT​TAC​CAT​CAA​GGC​TCG​G
GAPDH	Forward	GAC​AAC​AGC​CTC​AAG​ATC​ATC​AG
	Reverse	GTG​GCA​GTG​ATG​GCA​TGG​A

### Western Blot Analysis

Proteins were extracted from intestinal tissue or cultured NCM460 cells. Protein concentration of each sample was determined by BCA detection kit (Beijing, Shanghai). The precooled RIPA cell lysate was added, and the protein concentration in the tissue was determined using the BCA kit. Sample total proteins were isolated with 10%SDS-PAGE, then transferred to polyvinylidene difluoride (PVDF) membranes and incubated with appropriate antibodies. The protein was visualized using the chemical illuminating agent ECL. Protein bands in each group were exposed, and gray values of protein in each group were calculated using ImageJ. The software was used to analyze the image and determine the gray value of the protein bands, with GAPDH as the internal reference.

### Severity Evaluation of Sepsis

The intestinal tissue of the CLP model was fixed in 4% paraformaldehyde, the sample was dehydrated and embedded in paraffin wax. The embedded intestinal tissue was cut into 2–3 mm sections and stained with hematoxylin for 10 min and eosin for 2 min at room temperature. The pathological changes of the sections were observed by optical microscope, and the intestinal mucosal injury was evaluated by Chiu’s score grading. (Chen et al., 2020) The mouse CLP model was evaluated using a Shrum’sscore, (Mathiasen and DiCamillo, 2010) which included the mouse’s appearance, level of consciousness, activity, response to touch and auditory stimuli, eyes, breathing rate and quality, with scores ranging from 0 to four for each item indicated above. The higher the score, the more severe the sepsis.

### Immunofluorescence and Immunohistochemistry Staining

Paraffin section was dewaxed. The cells were permeabilized with PBS containing 0.5% Triton X-100 for 10 min, and then, The primary antibodies ZO-1(ABclonal, A0659, 1:100), Occludin (ABclonal, A2601, 1:100), claudin-1(ABclonal, A2196, 1:100) were added to the cells and incubated overnight at 4°C. The cells were rinsed with PBS 3 times for 5 min each time. Then, the cells were placed in the secondary antibody with darkness for 1 h at room temperature. After being cleaned with PBS again, the sections were developed with DAB, and then the cells were restained with hematoxylin solution and sealed (Beijing Zhongshan). The immunohistochemical slides were imaged with a light microscope (Diaphot 300, Nikon). Each sample was observed in five fields and the target protein signal was stained with brown. The number of positive cells/total cells was calculated using image analyzer (Image-Pro Plus 5.1, MediaCybernetics, MD, United States). For IF staining, we preincubated dewaxed sections with PBS3% BSA for half an hour. Then, we used 4’,6-diamidino-2-phenylindole to counterstain these sections, and after that, we washed and installed these sections in antifade medium. We developed it using a fluorescence microscope (NIKON eclipse T2000-U).

### Enzyme-Linked Immunosorbent Assay

The serum levels of tumor necrosis factor-α and interleukin-6 in mice were determined by enzyme-linked immunosorbent assay (ELISA) according to the instructions of the ELISA kit. In short, the sample was first diluted and the sample and antibody were added to the enzyme-labeled plate. The board was washed with washing solution 5 times before adding coloring solution and stop solution. Finally, a microorifice plate reader was used to measure the absorbance at 450 nm.

### Oxidative Stress Injury Detection

The supernatant was obtained after homogenization of intestinal tissue. SOD (Beyotime, S0103), MDA(Beyotime, S0131S) and GSH(Beyotime, S0056) levels were measured according to the reagent seller’s instructions(Beyotime Biotechnology, Shanghai, China.). The content of reactive oxygen species (ROS) in intestinal tissue was detected by DHE fluorescent probe (Sigma-Aldrich, MAK143). The fresh intestinal tissue was embedded and cut into 7 μm slices. The sections were incubated with DHE solution at 37°C for 30 min in dark. Put the slide into PBS(PH7.4) and shake it 3 times for 5 min each time. The tissue was then incubated at room temperature with DAPI solution for 10 min. Finally, the slides were examined under a fluorescence microscope.

### Hematoxylin and Eosin Staining

HE staining was performed according to routine protocols. Briefly, after deparaffinization and rehydration, the tissue sections were stained with hematoxylin solution (ZSGB-BIO, China) for 5 min followed by five dips in 1% acid ethanol (1% HCl in 75% ethanol) and then rinsed in distilled water. It was then stained with eosin solution (ZSGB-BIO, China) for 3 min and followed by dehydration with graded alcohol and clearing in xylene. The installed slides were then checked and photographed using an LEICA DM3000 LED (Leica DMshare (v3), Germany).

### Statistical Analyses

All tests were carried out using SPSS 20.0 software. The experimental data were expressed as mean ± SD. Differences between the two groups were measured by *t*-test, and comparisons between the groups were evaluated by one-way analysis of variance (ANOVA). Statistical significance was defined as *p* < 0.05 and the graph was generated by GraphPad 8.0.

## Results

### *In vitro* Cytotoxicity of Emodin

**FIGURE 1:**
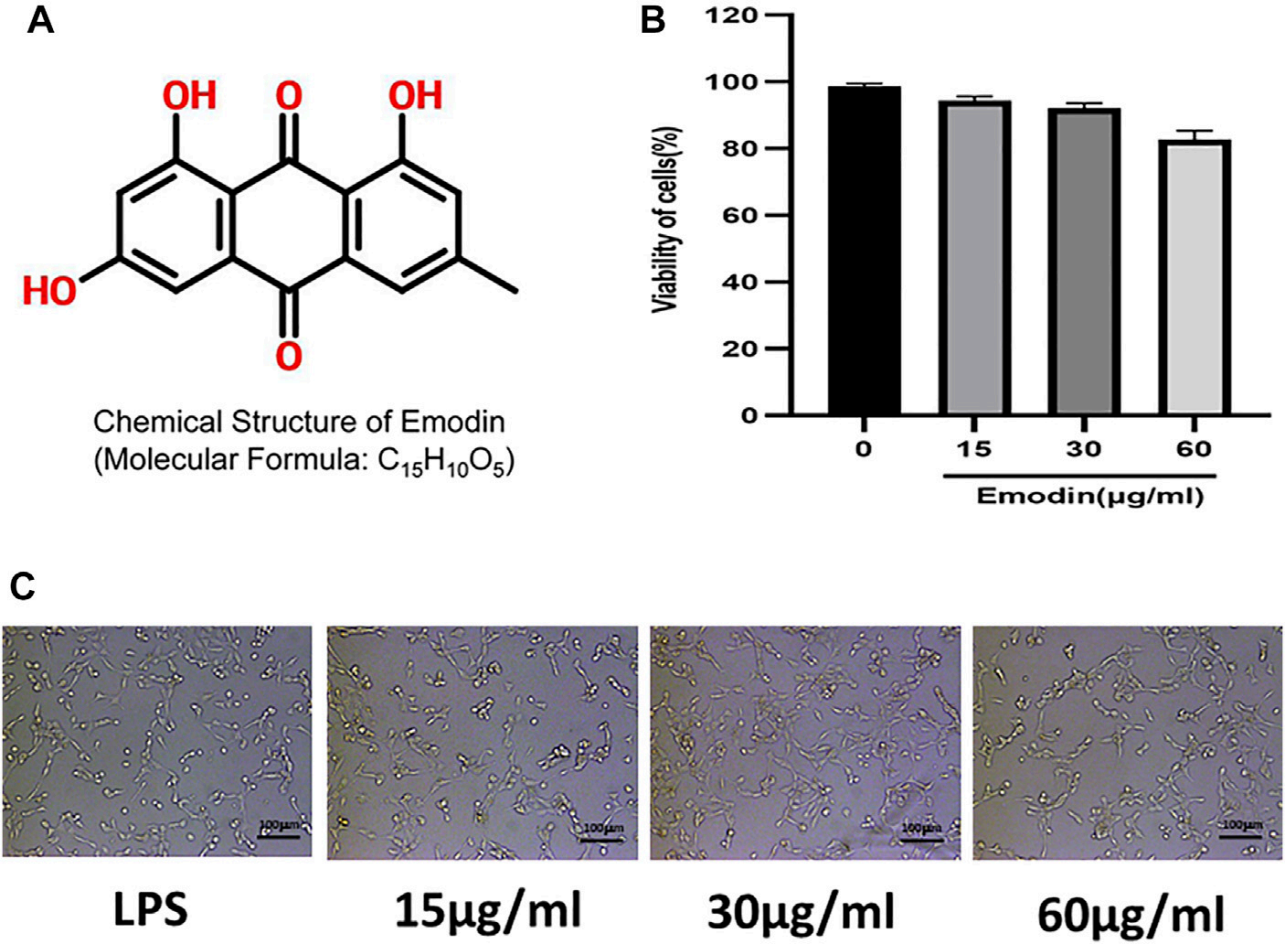
**(A)** The chemical structure of emodin. **(B)** A CCK-8 assay of NCM460 cells viability after emodin treatment. **(C)** Morphology of NCM460 cells treated with different concentrations of emodin for 12 h.(scale bar: 100 μm)

In order to determine the effect of emodin on the viability of NCM460 cells, cell counting kit-8 (CCK-8) was used to detect the viability of cells treated with different concentrations of emodin (Figures 1A,B). The results showed that the cell viability was 75% when the emodin concentration was 60μg/ mL. Therefore, we chose a high concentration of 60μg/ml, a medium concentration of 30μg/ml and a low concentration of 15 μg/ml. In addition, the morphological changes of NCM460 cells treated with different concentrations of emodin were not significant (Figure 1C).

### Effect of Emodin on VDR and Its Downstream Molecules After VDR Knockout

**FIGURE 2:**
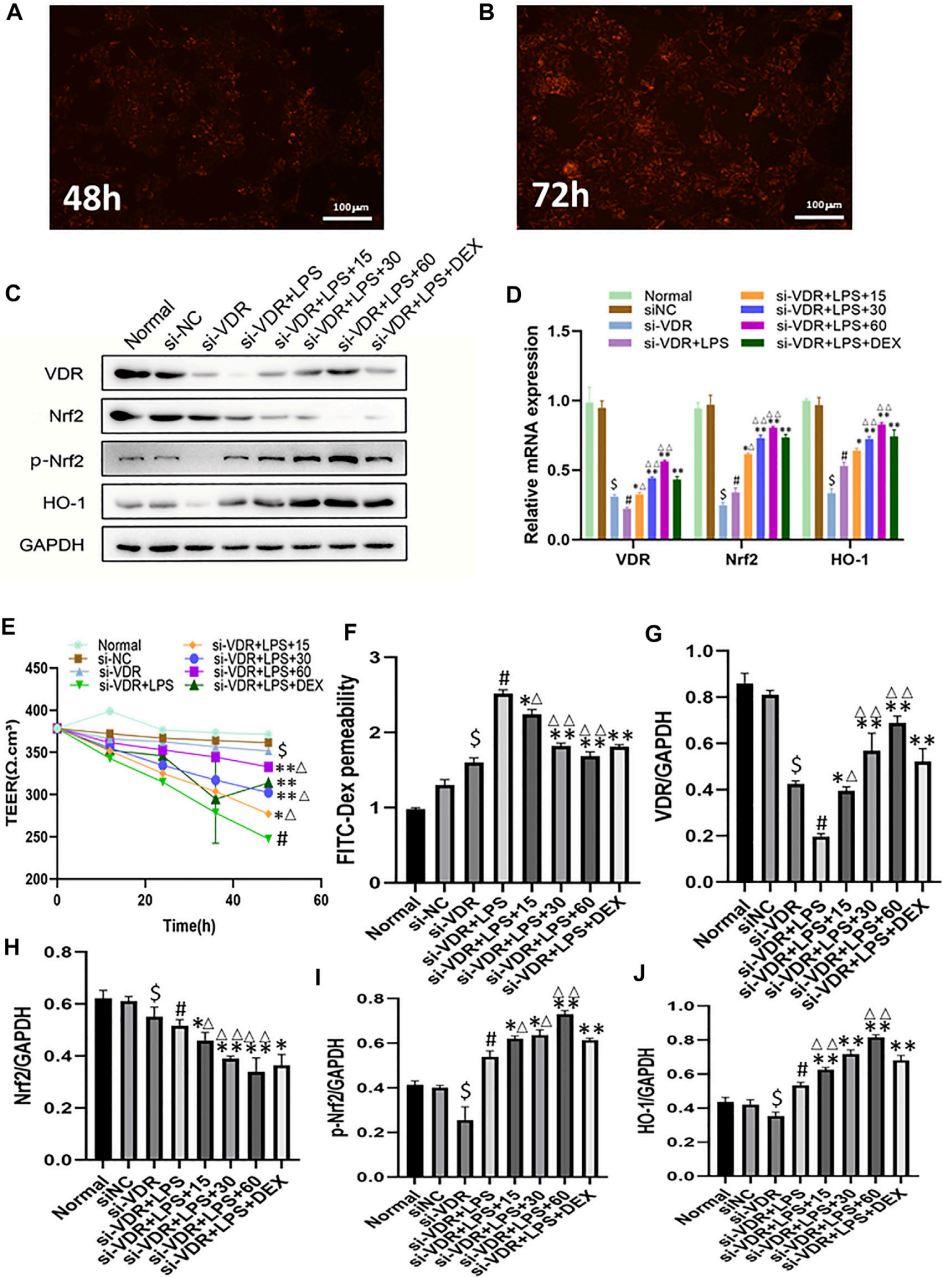
**(A-B)** The expression of VDR was observed with a fluorescence microscope after siRNA was introduced into NCM460 cells for 48 h and 72 h **(C)**The protein levels of VDR, Nrf2, *p*-Nrf2, HO-1 molecules were detected by western blotting. **(D)** The mRNA levels of above molecules were detected by RT -PCR. **(E–F)** Transepithelial resistance (TEER) and FITC-Dextran 4 kDa flux were measured to assess monolayer permeability. **(G–J)** Quantitative results of VDR and its downstream protein expression. ^$^ *p* < 0.05 compared with the normal group; ^#^ *p* < 0.05 vs si-VDR group; ^∗^ *p* < 0.05,^∗∗^ *p* < 0.01 vs si-VDR + LPS group; ^∆^ *p* < 0.05, ^∆∆^ *p* < 0.01, compared with the DEX group. si-VDR: VDR was knocked down in NCM460 cells by siRNA. si-NC: SiRNA negative control group.

In order to accurately down-regulate the expression of VDR, we used siRNA to interfere with VDR in NCM460 cells, and detected fluorescence signals 48 and 72 h after si-RNA transfection into cells (Figures 2A,B). The emodin, DEX and model groups were stimulated with LPS (1 g/ml) for 2h, and then emodin (60, 30 or 15 μg/ml) and DEX were added. As shown in Figures 2C,D, we observed that there was no statistical significance between the siRNA-negative control group and the normal control group (*p* > 0.05). Compared with the normal group, the mRNA and protein levels of VDR, Nrf2 and HO-1 in si-VDR group were significantly decreased (*p* < 0.01). Compared with si-VDR group, the levels of VDR and its downstream Nrf2 in si-VDR-LPS group were decreased, while the level of HO-1 was increased.(*p* < 0.01). Compared with si-VDR-LPS group, emodin and Dex groups showed significantly increased mRNA and protein expression levels of VDR, Nrf2 and HO-1. In addition, the mRNA and protein levels of VDR, Nrf2 and HO-1 in emodin (30 and 60 μg/mL) groups were significantly different from those in DEX group (*p* < 0.05 or *p* < 0.01). As shown in Figures 2E,F, the TEER of monolayer cells at different times was measured to evaluate monolayer permeability. When NCM460 cells were incubated with LPS (1 g/ ml), the TEER and the paracellular permeability of FITC value showed a rapid decline and reached its lowest level 48 h later, while the addition of emodin and dexamethasone improved the decrease, with 60 μg/ mL having the strongest improvement.As shown in Figures 2G–J, The graphs showed quantification of VDR and its downstream protein expression.

### Emodin Alleviates Intestinal Barrier Dysfunction After VDR Knockout

**FIGURE 3:**
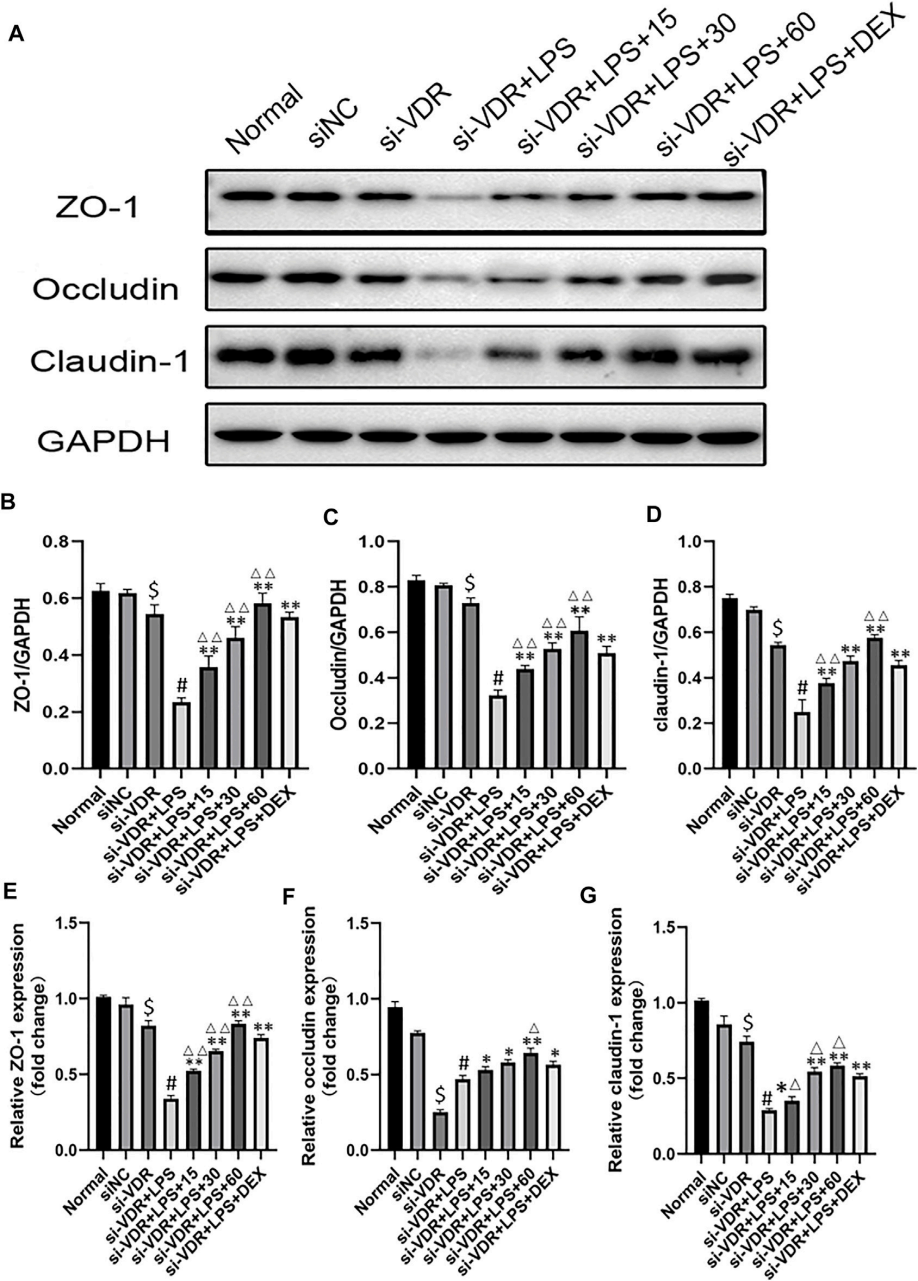
**(A-D)** The protein levels of ZO-1, Occludin and Claudin-1 molecules were detected by western blotting. **(E–G)** The mRNA levels of above molecules were detected by RT -PCR. ^$^ *p* < 0.05 compared with the normal group; ^#^ *p* < 0.05 vs si-VDR group; ^∗^ *p* < 0.05,^∗∗^ *p* < 0.01 vs si-VDR + LPS group; ^∆^ *p* < 0.05, ^∆∆^ *p* < 0.01, compared with the DEX group.si-VDR: VDR was knocked down in NCM460 cells by siRNA. si-NC: SiRNA negative control group.

In order to detect the protective effect of emodin on intestinal mucosa through the VDR/ Nrf2 /HO-1 pathway, we used siRNA to interfere with VDR in NCM460 cells. As shown in Figure 3A, we observed that there was no statistical significance in the expression level of mucosal molecules between the siRNA-negative control group and the normal control group (*p* > 0.05). Compared with the normal group, the expression levels of ZO-1, Occludin and Claudin-1 in si-VDR group were decreased. Compared with si-VDR group, the expression level of mucosal molecules in si-VDR-LPS group was significantly decreased (*p* < 0.01). Compared with si-VDR-LPS group, emodin and DEX groups showed significantly increased mRNA and protein expression levels of ZO-1, Occludin and Claudin-1. In addition, the mRNA and protein levels of ZO-1, Occludin and Claudin-1 in emodin (30 and 60 μg/mL) group were significantly different from those in DEX group (*p* < 0.05 or *p* < 0.01). Figures 3B–G

### Emodin Improved Inflammation and Oxidative Stress Levels in Mice

**FIGURE 4:**
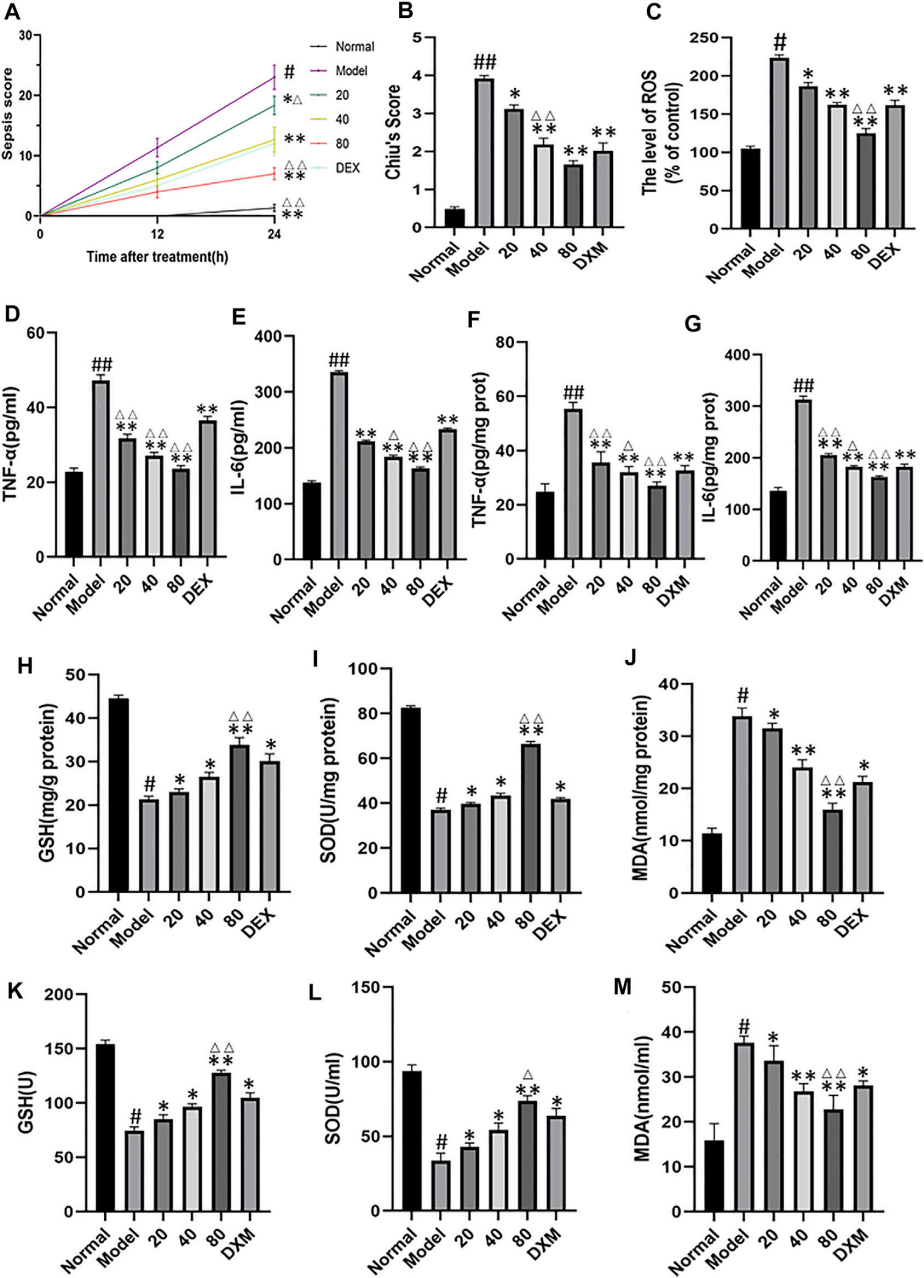
**(A)** Effect of emodin on sepsis score in septic mice. **(B)** Chiu’s score was used to evaluate pathological injury of intestinal tissue. **(C)** ROS levels in intestinal tissue were detected with DHE probe. **(D–G)** Effect of emodin on the expression of TNF-α and IL-6 according to ELISA. **(H–M)** The levels of malondialdehyde (MDA), superoxide dismutase (SOD), and glutathione (GSH) in micetissue were measured using the oxidative stress detection kit. Data are presented as the mean ± standard error of mean (*n* = 8 in each group). ^#^ *p* < 0.05, ^##^ *p* < 0.01 vs normal group; ^∗^ *p* < 0.05,^∗∗^ *p* < 0.01 vs model group; ^Δ^P< 0.05, ^ΔΔ^P< 0.01, compared with the DEX group.

The shrum score was used to evaluate the severity of sepsis in mice. The results showed that 12 h after caecal ligation and puncture, the mice began to show some disease symptoms. At this point, the average scores of the model group and the low-emodin, medium-emodin and high-concentration emodin groups were 11.3, 8, 6 and 4, respectively, and there was no significant statistical difference between the groups. At 24 h postoperatively, mice in the model group showed a significantly higher sepsis score compared to the normal group with a score of 1. Compared with CLP mice, emodin administration at low, medium and high concentrations significantly reduced the mean sepsis score. The scores of the three groups were 18.3 (*p* < 0.05), 12.6 (*p* < 0.01) and 7(*p* < 0.01), respectively. (Figure 4A) Chiu’s score was used to evaluate the pathological injury of intestinal tissue. The higher the score was, the more serious the injury was. The Chiu’ score of model group was significantly higher than that of control group. Compared with model group, the scores of low, medium and high emodin groups were lower and lower. (Figure 4B)The expression of TNF-α and IL-6 in serum and tissue were detected by enzyme linked immunosorbent assay. As shown in Figures 4D–G levels of TNF-α and IL-6 in the model group were significantly higher than those in the normal group (*p* < 0.01). Compared with the model group, the expression of TNF-α and IL-6 in the emodin intervention group was significantly decreased (*p* < 0.05 or *p* < 0.05), and emodin could inhibit the expression of TNF-α and IL-6. ROS levels in intestinal tissue were detected by DHE probe (Figure 4C), and emodin administration could reduce the production of reactive oxygen species. The levels of malondialdehyde (MDA), superoxide dismutase (SOD), and glutathione (GSH) in mice serum and tissue were measured using the oxidative stress detection kit. As expected, MDA levels were significantly reduced and GSH and SOD levels were significantly increased after emodin administration (Figures 4H–M). The above results on oxidative stress were consistent with the changes in intestinal histopathology, suggesting that emodin had an anti-oxidative stress effect.

### Emodin Alleviates Intestinal Barrier Dysfunction Caused by Cecal Ligation and Puncture Model

**FIGURE 5:**
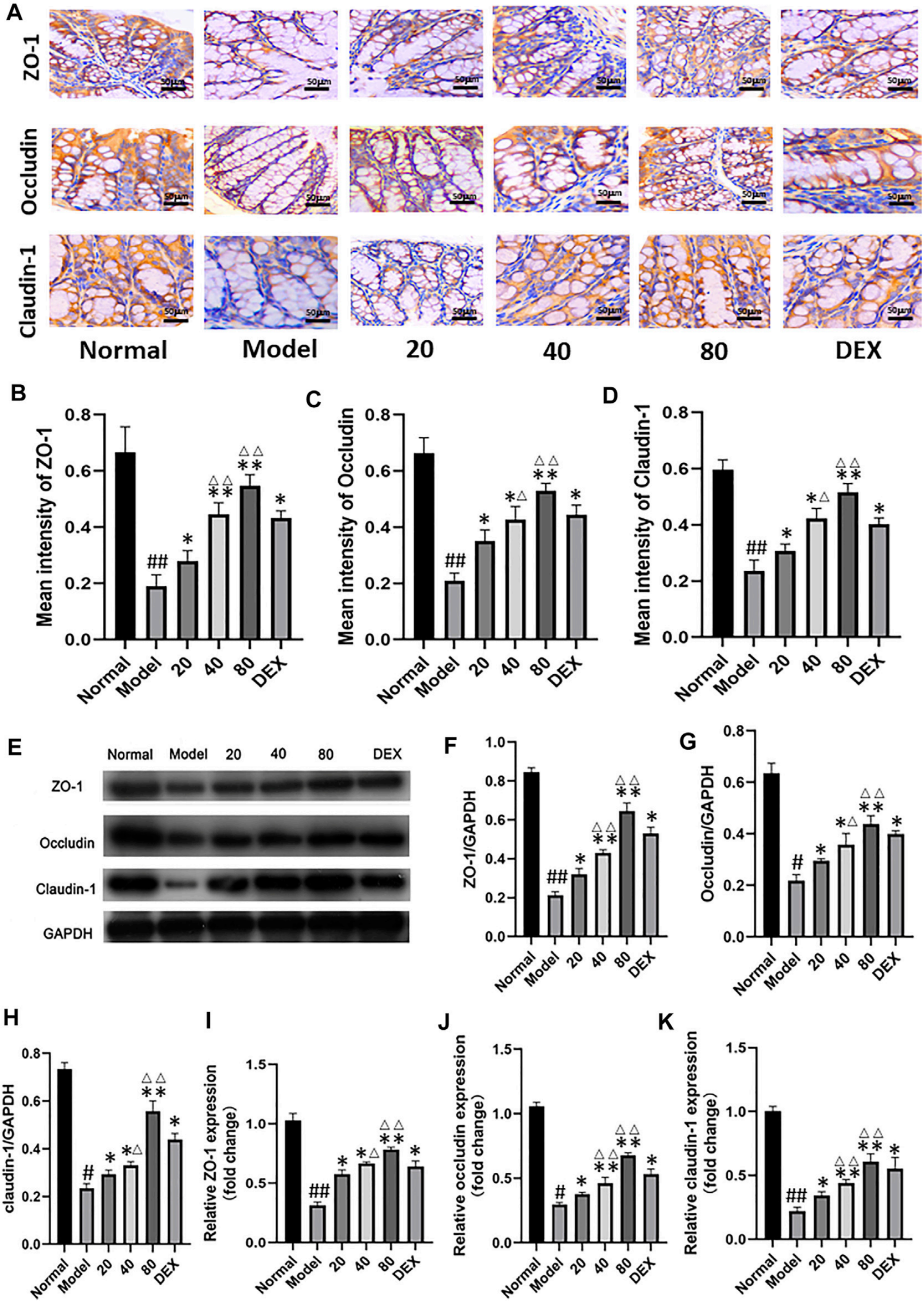
**(A)** Immunohistochemical analysis of ileum tissue. (magnification ×200). **(B–D)** Immunohistochemical results of tight junction protein were quantified. **(E)** Western blotting analysis of TJ proteins (ZO-1, Occludin, and Claudin-1). **(F–H)** Representative protein levels of ZO-1/GAPDH, Occludin/GAPDH, and Claudin-1/GAPDH (n = 3). **(I–K)** Relative mRNA of ZO-1, Occludin and Claudin-1 in mice ileum tissue were measured by qPCR (n = 8). Each bar represents mean ± SD , ^#^ *p* < 0.05, ^##^ *p* < 0.01 vs normal group; *p < 0.05, **p < 0.01 vs model group; ^Δ^P< 0.05, ^ΔΔ^P< 0.01, compared with the DEX group.

To assess intestinal barrier dysfunction in sepsis, we collected immunohistochemical images of mouse ileum tissue (Figure 5A). By quantifying the immunohistochemical results of mucosa-related molecules, the expression of ZO-1, occluding and claudin-1 in the model group decreased compared with the normal group. Compared with model group, the molecular expression of emodin group increased gradually with the increase of concentration. (Figures 5B–5D) Compared with the normal group, we observed a decrease in intestinal epithelial villi in the model group, which was relieved with emodin administration. Tight-junction (TJ) proteins, mainly occludins, claudins, and small band occludins (ZO), help to maintain intestinal barrier function. By Western blot and real-time quantitative polymerase chain reaction (Figures 5E–H), we found that the protein levels of ZO-1, occludin and claudin-1, and were decreased in CLP-induced septic intestinal tissue, and the expression of these proteins was increased after emodin administration (*p* < 0.01). We further confirmed this trend by measuring the expression levels of ZO-1, Occludin and Claudin-1 and by quantitative polymerase chain reaction (Figures 5I–K). Our results suggest that emodin alleviates CLP-induced intestinal barrier dysfunction.

### Effect of Emodin on VDR and Downstream Molecules After Cecal Ligation and Puncture Model Stimulation

**FIGURE 6:**
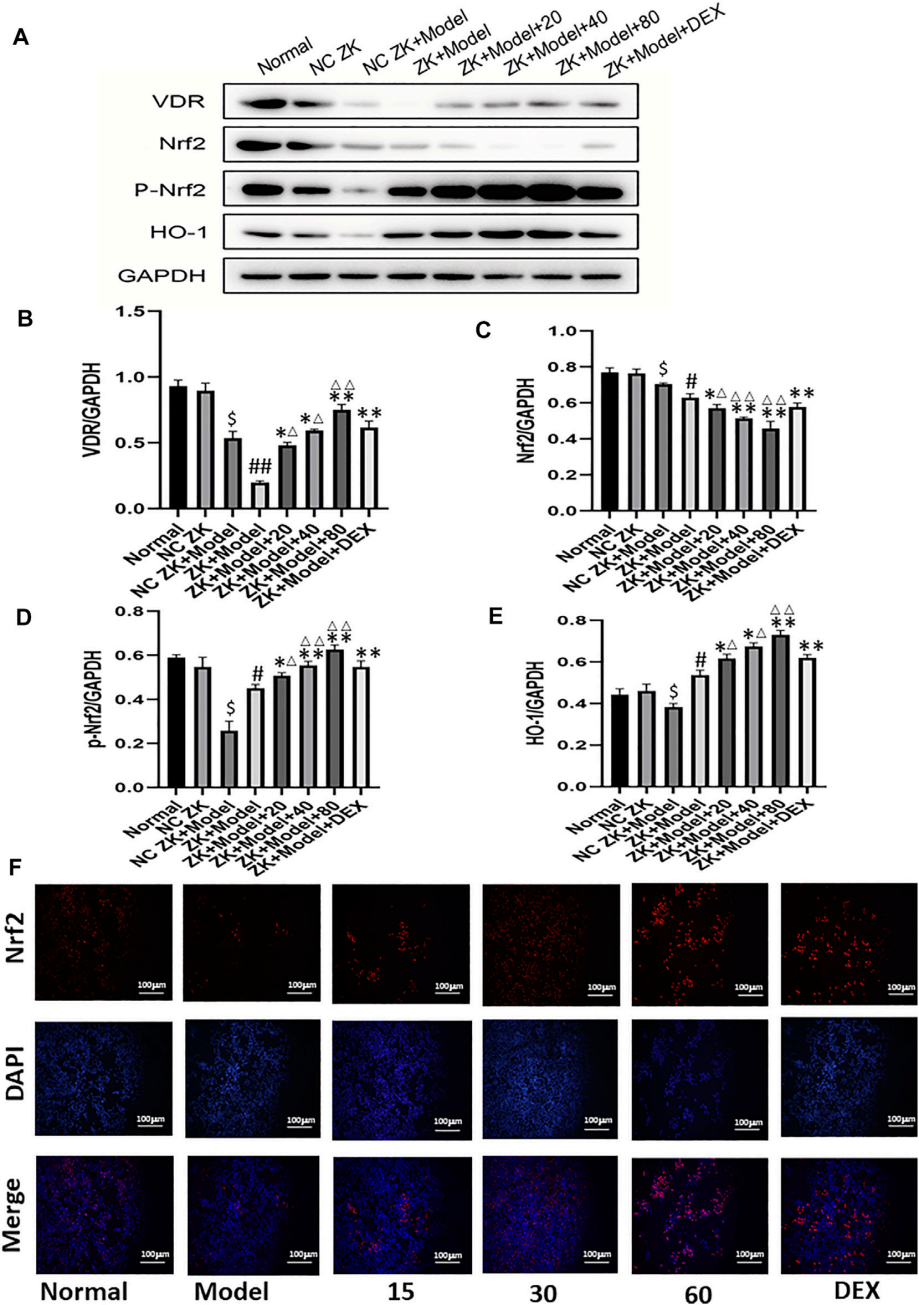
**(A–E)** The protein levels of VDR, Nrf2, *p*-Nrf2, HO-1 molecules were detected by western blotting.**(F)** Immunofluorescence was used to detect the Nrf2 nuclear translocation of cells. ^#^ *p* < 0.05, ^##^ *p* < 0.01 vs normal group; ^∗^ *p* < 0.05,^∗∗^ *p* < 0.01 vs model group; ^Δ^P< 0.05, ^ΔΔ^P< 0.01, compared with the DEX group.

As shown in Figure 6A, gene and protein levels of VDR were significantly decreased in the model group compared with the normal group. Meanwhile, CLP and emodin had opposite effects on VDR expression. In addition, the protein expression levels of downstream molecules P-NrF2 and HO-1 in model group were significantly increased (*p* < 0.05), while the protein expression of Nrf2 was decreased. (*p* < 0.05). After 24 h treatment with different concentrations of emodin, the expression of VDR increased in low, medium and high concentrations of emodin groups compared with model group. More importantly, the levels of downstream Nrf2 and HO-1 in emodin (80 mg/ kg) group were significantly higher than those in DEX group (*p* < 0.01). Emodin treatment significantly increased the expression of VDR and downstream molecules and promoted nucleation of Nrf2 molecules after CLP stimulation. (Figures 6B–F)

### Emodin Alleviates Cecal Ligation and Puncture Model-Induced Liver and Lung Injury

**FIGURE 7:**
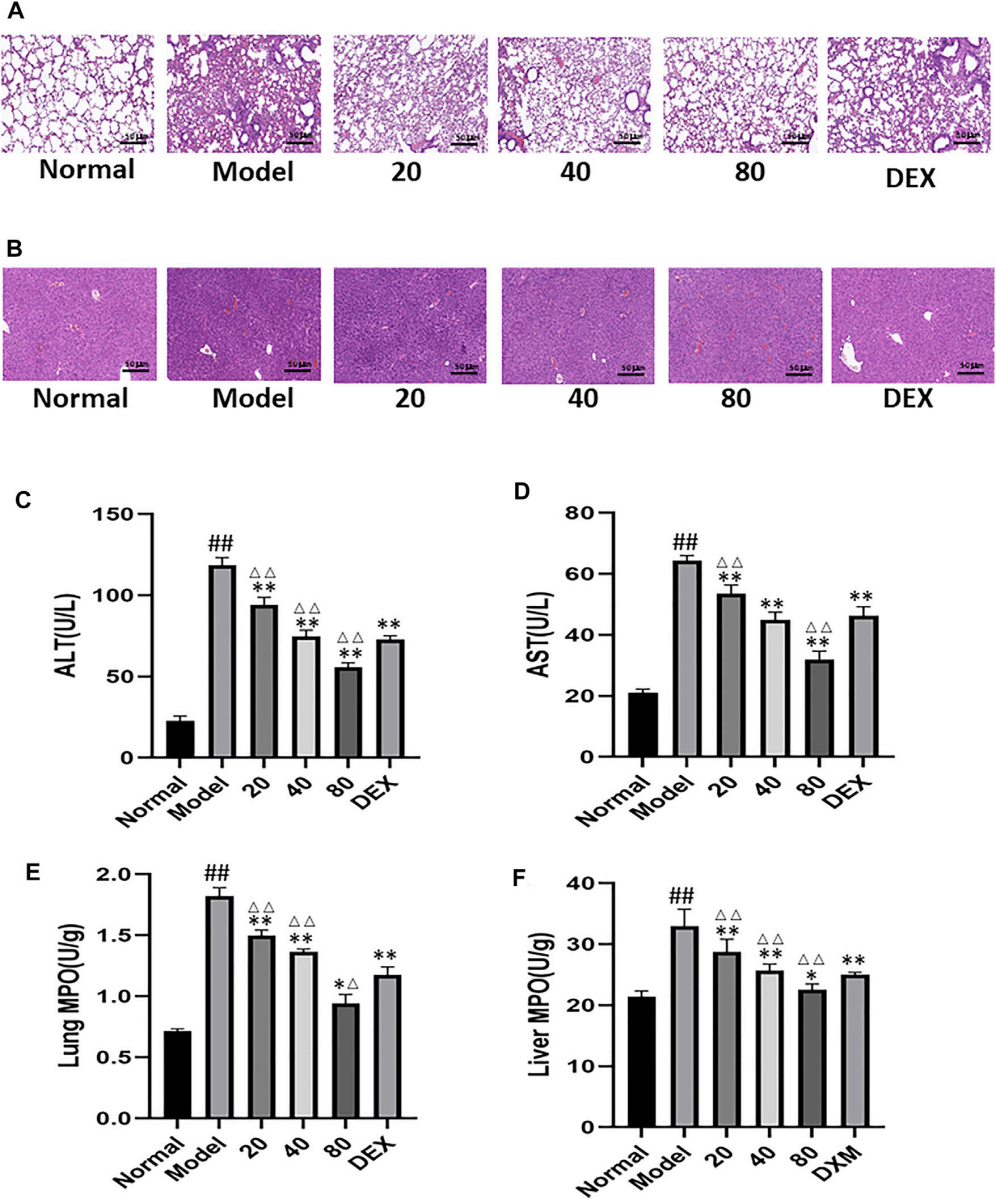
**(A)** Representative images of H&E-stained lung sections from mice. (original magnification ×200).**(B)** Representative images of H&E-stained hepatic sections from mice. **(C–D)** Serum ALT levels and AST levels. **(E,F)** MPO activity in the lung and liver. ^#^ *p* < 0.05, ^##^ *p* < 0.01 vs normal group; ^∗^ *p* < 0.05,^∗∗^ *p* < 0.01 vs model group. ^Δ^P< 0.05, ^ΔΔ^P< 0.01, compared with the DEX group.

Emodin can alleviate CLP-induced distal organ damage. CLP is always associated with distal organ damage, which plays a crucial role in prognosis. Therefore, the liver and lung tissue injuries were examined to assess the protective effect of emodin on remote organ injuries. As shown in Figures 7A,B, emodin significantly alleviated CLP-induced liver and lung histological damage. Compared with the blank group, the levels of alanine aminotransferase(ALT) and aspartate aminotransferase(AST) were significantly increased in the model group. After emodin treatment, ALT and AST levels were significantly reduced (Figures 7C,D). Similarly, neutrophilic infiltration in the lung and liver (as shown by MPO activity) was significantly reduced after emodin administration (Figures 7E,F). These results suggest that emodin can improve remote liver and lung injury caused by CLP.

## Discussion

Studies have shown that emodin is a potential candidate drug for the treatment of sepsis associated intestinal mucosal injury (Ning et al., 2017; Tan et al., 2020). However, the underlying mechanism of emodin’s pharmacological activity is still not fully understood. This study investigated the possible molecular mechanisms and protective effects of emodin on sepsis related intestinal mucosal injury *in vivo* and *in vitro*. The results of this study clearly showed that emodin could significantly up-regulate the expression levels of Nrf2 and HO-1 molecules in VDR and its downstream pathways, reduce the levels of inflammatory cytokines TNF-α and IL-6, and improve oxidative stress levels. Finally, emodin administration significantly inhibited inflammatory response and oxidative stress, intestinal mucosal damage, and distal lung and liver tissue damage.

Excessive production of inflammation and oxidative stress is the main reason for the occurrence and development of sepsis. (Lee and Lee, 2014) Therefore, it is particularly important to effectively control the development of inflammation and oxidative stress in the early stage. The pharmacological effects of dexamethasone are concentrated in anti-inflammatory (Yu et al., 2019), anti-endotoxin (Yang et al., 2019), immunosuppressive (Hoppstädter et al., 2019; Kim et al., 2020), and anti-shock (O'Connor et al., 2003). Although some studies have shown that dexamethasone is effective in improving inflammation and regulating immune function (Bao and Dai, 2020; Fang et al., 2021), long-term use of glucocorticoids is associated with a series of side effects, including obesity, hypertension, osteoporosis, etc., (Son et al., 2017; Herrera et al., 2020; Xu et al., 2020) Therefore, it is important to find effective drugs with minimal side effects for the treatment of sepsis associated intestinal mucosal injury.

In previous studies, emodin can play a protective role in intestinal mucosa. However, no studies have reported that emodin can act on VDR molecules and activate the downstream Nrf2/HO-1 pathway, thereby reducing inflammation and oxidative stress and protecting intestinal mucosal damage. VDR is an important regulator of intestinal cell proliferation, barrier function and immunity. (Bakke and Sun, 2018; Fakhoury et al., 2020) Second, VDR can activate the SIRT1/ NRF-2 pathway and inhibit the NF-κB pathway, which has a protective effect on tumor necrosis factor-α induced intestinal barrier function impairment. (Yao et al., 2019) Therefore, the VDR/ NRF2 /HO-1 signaling pathway, a potentially important therapeutic target, has a prominent role in sepsis associated intestinal mucosal injury.

In our study, we provide evidence that emodin can protect intestinal mucosa through the VDR/ NRF2 /HO-1 signaling pathway *in vitro* and *in vivo*. *In vitro*, the VDR signaling pathway was activated by lipopolysaccharide. Compared with the model group, the mRNA and protein expression levels of VDR, Nrf2, p-Nrf2 and HO-1 in emodin group were significantly increased. Then, we used the synthetic VDR-siRNA to knock down the expression of VDR. When VDR was knocked down and when LPS activated NCM460 cells, emodin increased the mRNA and protein expression levels of VDR, Nrf2, p-Nrf2, and HO-1.

In animal experiments, the results showed that emodin could protect mice from intestinal mucosal injury induced by cecal ligation and puncture by inhibiting inflammatory and oxidative stress responses through VDR. CLP is the most commonly used experimental model for sepsis. The development of sepsis involves recruitment and activation of inflammatory cells, oxidative stress, and a cascade of cascading reactions that lead to increased intestinal epithelial cell permeability and intercellular barrier dysfunction. Our results showed that intestinal tight junction protein expression was increased in emodin group compared with model group. The expression of tumor necrosis factor-α and interleukin-6 decreased, the levels of malondialdehyde decreased significantly, and the levels of glutathione and superoxide dismutase increased significantly.

Our study had some limitations. Sepsis—associated intestinal mucosal injury in an animal model in our experiment is a rapid onset process. Therefore, in order to better understand CLP-induced intestinal mucosal injury, more work should be done to clarify the detailed mechanisms.

## Conclusion

Emodin protects sepsis related intestinal barrier damage through the VDR/ NRF2 /HO-1 signaling pathway. VDR may be a potential therapeutic target for emodin in the treatment of intestinal barrier injury

## Data Availability

The raw data supporting the conclusion of this article will be made available by the authors, without undue reservation.
